# Multistage Extraction of Star Anise and Black Pepper Derivatives for Antibacterial, Antioxidant, and Anticancer Activity

**DOI:** 10.3389/fchem.2021.660138

**Published:** 2021-05-14

**Authors:** Helin Li, Xiaoyu Wu, Xin Li, Xiaobing Cao, Yanjun Li, Huaru Cao, Yongzhi Men

**Affiliations:** ^1^Zhejiang Provincial Key Laboratory of Chemical Utilization of Forestry Biomass, Zhejiang A & F University, Hangzhou, China; ^2^Co-Innovation Center of Efficient Processing and Utilization of Forest Resources, Nanjing Forestry University, Nanjing, China; ^3^Shanghai General Hospital, Shanghai Jiao Tong University School of Medicine, Shanghai, China; ^4^Department of Surgical Oncology, Affiliated Hospital of Nanjing University of Chinese Medicine, Nanjing, China; ^5^Institute of Technical and Macromolecular Chemistry, RWTH Aachen University, Aachen, Germany

**Keywords:** natural product, multistage extraction, antibacterial, antioxidant, anticancer

## Abstract

Recently, natural resources have attracted considerable interest for their applications in food security and human health problems. Traditional natural spices, such as star anise and black pepper, played important roles in the pharmaceutical and food industries due to their strong pharmacological activity, antioxidant potential and rare complications. In order to achieve biomasses from the natural product with multiple bioactivities, we developed the multistage extraction method to extract and separate various bioactive compounds from these natural plants. Our work demonstrated that various bioactive-rich extractives were achieved using steam distilled- or oxidative-extraction methods with high extraction yields and purity. Furthermore, the extractives in each step can be used not only as bioactive compounds, but also as a resource to further prepare different derivatives during the next extractive step, providing biomass-saving to a great extent. The extractives obtained with high yields and purities (>82%) were identified by ^1^H NMR, ^13^C NMR, FTIR, UV-vis, fluorescence spectroscopy, and high-performance liquid chromatography (HPLC). Moreover, these biomasses display potent antibacterial activities against some types of microorganisms such as *S.aureus, S.pyogenes, E.coli*, and *S.typhi* with a lowest MIC of 400 μg/ml for the development of antibacterial agents, significant antioxidant activity as the natural antioxidant for enhancing food shelf-life, and excellent anticancer activity that induces significant cancer cell apoptosis. This work showed the different multistage extracts from natural products, which enable them to be applied in the fields of the pharmaceutical industry and the food industry.

## Introduction

Recently, food security and human health have become global threats and attracted worldwide concerns (Ruiz-Capillas and Herrero, 2019; Thompson and Darwish, 2019; Yousefi et al., 2019). In the food industry fields, severe foodborne illness and intoxications have grown to be a widespread problem and risked public health (Hui et al., 2020; Jing et al., 2020; Sandra and Elaine, 2020). Moreover, extending the shelf-life of food products is also an urgent requirement for food quality (Olatunde and Benjakul, 2018). For human health problems, the increased number of people suffering from cancer and multi-resistant bacterial infections have been seen as the major causes of death (Li et al., 2016b, 2017b; Li X. et al., 2019). In order to solve the crisis, antioxidants are capable of quenching reactive free radicals, and thus inhibiting peroxidation and extending the food storage process. Antibacterial and anticancer agents can be used against various infections and diseases as future chemotherapeutic drugs (Xing et al., 2020; Zhong et al., 2020; Li et al., 2021c). Among these bioactive compounds, each of them can be capable of acting as one active agent, but without multiple actions. It is urgent to develop new therapeutic agents with multiple activities against microbial infections and cancer to administrate this pathogen for human diseases and the food industry (Li et al., 2017a, 2021a,b).

Natural resources have attracted considerable interest by many scientists on the natural extracts identification due to their potential applications in treating human health problems and enhancing food security (Li et al., 2014, 2015; Liu J. et al., 2020). Traditional spices and food flavoring agents, such as star anise and black pepper, date back several thousand years (Liu Q. et al., 2020; Pei et al., 2020; Wang et al., 2021). They played important roles in clinical therapy and the food industry due to their beneficial medical use (Moghaddasi et al., 2018; Kamoun et al., 2019; Li H. et al., 2019), strong pharmacological activity (Abukawsar et al., 2018; Li et al., 2020; Yu et al., 2020), antioxidant potential (Liu et al., 2008), and low cost (Destro et al., 2019; Orrillo et al., 2019; Ding et al., 2020).

Among these natural resources, star anise has been widely used as a common spice in culinary as well as medical plants (Patra et al., 2020). The pharmaceutical ingredients of star anise have been studied and some kinds of extractives have been used in medicine. Anise oil, a star anise extract, exhibited significant antifungal, antioxidant, and antimicrobial potential, which are mainly due to the high contents of more than 80% trans-anethole (Aly et al., 2016). Star anisic acid (4-methoxybenzoic acid) (ANISA), an extractive of star anise, protects the liver against the hepatotoxicity induced by carbon tetrachloride (CCl_4_) and paracetamol (Pcl) (Czarnecka et al., 2018; Lin et al., 2018). Previous studies have suggested that ANISA played a role in the prevention and treatment of cancer through affecting the activity of cyclooxygenase (COX-2), a mediator in tumorigenesis, due to their potential for the synthesis of a variety of pharmaceutical substances, such as chloral, an anticonvulsive agent, and phentobarbital for the treatment of refractory status epilepticus (RSE) (Tao et al., 2014). In addition, shikimic acid (3, 4, 5-trihydroxy-1-cyclohexene-1-carboxylic acid), an important raw material for the manufacture of oseltamivir phosphate (Tamiflu^®^), can be extracted from the fruits of star anise. Oseltamivir phosphate (Tamiflu^®^) is an oral prodrug to treat patients infected by Influenza virus A (Shin et al., 2017). Therefore, star anise biomass showed a great potential in the pharmaceutical industry as well as in the food industry.

Another most popular spice is black pepper, obtained from *Piper nigrum* L. belonging to the family Piperaceae (Tran et al., 2019). It can be applied in biomedicine for pain relief (Chavarria et al., 2016), muscular pains (Vichiansiri et al., 2020), flu, and fevers (Ahmad et al., 2020), especially in traditional Chinese and Indian medicines. Black pepper contains a wide variety of bioactive compounds in its oleoresins including essential oils and the alkaloid piperine (Dutta and Bhattacharjee, 2017; Hu et al., 2019; Wang et al., 2020). The biological activities of black pepper are attributed to its piperine content, which is the extraction of alkaloid from black pepper. As the major constituent of pepper, piperine exhibited various pharmacological activities, e.g., antifungal (Phuna et al., 2020), antipyretic (Haq et al., 2021), anti-diarrhoeal (Goyal, 2017), analgesic (Chen et al., 2017), and anti-inflammatory activities (Li et al., 2016a; El-Ghazaly et al., 2020). However, the pharmaceutical activities and therapeutic properties of piperine are limited due to its poor water solubility. Therefore, this chemical substance needs to meet the demands for improving its poor dissolution in high therapeutic doses.

Due to the fact that the diverse biomass extractives of raw material, e.g., star anise and black pepper, possessed potent therapeutic efficacy, antioxidant, and antimicrobial activities, a number of approaches were introduced previously, such as hot water extraction (Hu et al., 2020), hydro-distillation (Ibrahim et al., 2017), steam-distillation (Nasrollahi et al., 2019), solvent extraction (Chaves et al., 2020), supercritical fluid CO_2_ extraction (Patra et al., 2020), and microwave-assisted extraction (Sun et al., 2020), to isolate and extract the bioactive components from these natural resources.

**Scheme 1:**
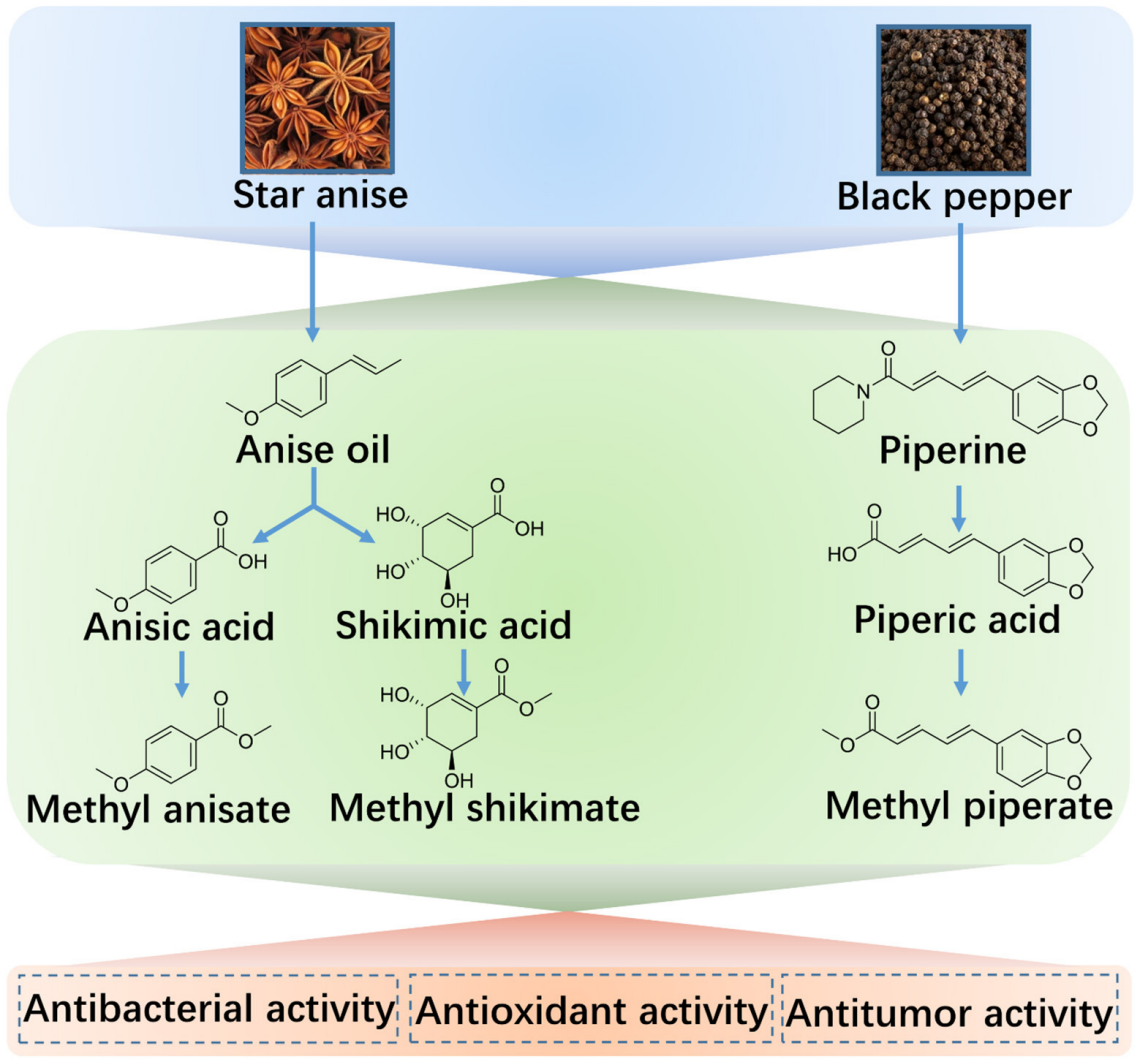
Schematic illustration of star anise or black pepper extractives and their applications.

Based on the promising therapeutic effects of these extractives, we developed the multistage methods to meet the requirements for the reproductive potential of the bioactive compounds from natural plants, star anise and black pepper. Depending on these reproductive technologies, five components (anise oil, anisic acid, methyl anisate, shikimic acid, methyl shikimate) of star anise and three ingredients (piperine, piperic acid, methyl piperate) of black pepper were prepared with a high yield by a multistage extraction-esterification method. Moreover, the extractives from the first stage can be applied as the bioactive agents and can also be used as the suppliers for the next extraction steps (Scheme 1). These extractives were characterized by 1HNMR, 13CNMR, FTIR, UV-vis, fluorescence spectroscopy, and HPLC, and the yields and purities were analyzed. Moreover, the bioactivity of these compounds was evaluated, showing the marked antibacterial activity against microorganisms, antioxidant potential as the natural antioxidants for enhancing food shelf-life, and anticancer activities as chemotherapeutic drugs engaged in clinical trials. Therefore, the obtained extractives provide diverse applications in the food as well as the pharmaceutical industry.

## Materials and Methods

### Materials

Star anise fruits and black pepper fruits were bought from Jiangda South Road Vegetable Market, Nanchang City, China. Magnesium sulfate (MgSO_4_), potassium dichromate (K_2_Cr_2_O_7_), sulfonic type solid acid, potassium permanganate (KMnO_4_), hydrochloric acid (HCl), ethanol absolute, hydrogen peroxide solution (30 wt. % in H_2_O, H_2_O_2_), Iron(III) chloride (FeCl_3_), methanol, sodium carbonate (Na_2_CO_3_), hexane, formaldehyde, thionyl chloride (SOCl_2_), ethyl acetate, sodium hydroxide (NaOH), acetone, diethyl ether, potassium hydroxide (KOH), benzene, sodium bicarbonate (NaHCO_3_), *N,N*-Dimethylformamide (DMF), 1,1-diphenyl-2-picryhydrazyl (DPPH), and butylated hydroxytoluene (BHT) were obtained from Sinopharm Chemical Reagent Co., Ltd.

The microorganisms *Staphylococcus aureus* (ATCC 33591) (MRSA), *Streptococcus pyogenes* (ATCC 19615), *Escherichia coli* (ATCC 9637), *Salmonella typhi* (ATCC 14028) and *Pseudomonas aeruginosa* (ATCC 10145) were purchased from Jinuo Biomedical Technology Co., Ltd (Hangzhou, China).

MTT [3-(4,5-dimethylthiazol-2-yl)-2,5- diphenyl tetrazolium bromide] and C6 cells were bought from Cell Bank of Shanghai Institute of Biochemistry and Cell Biology, Chinese Academy of Sciences (Shanghai, China).

### Diverse Extractives of Star Anise

#### The Extraction and Purification of Anise Oil

The anise oil was extracted by hydro-distillation methods. In a round bottom flask, 100 g of dried star anise fruit was grinded into pieces, and then extracted with 800 mL of deionized water under 70°C for 24 h using a Clevenger type apparatus, in accordance with Politeo et al. (2006). The extracts were filtered and most water was then removed. The anise oil extracts were dried by adding anhydrous MgSO_4_. After settling down, the suspension was obtained as the pure anise oil.

#### Anisic Acid Extraction and Preparation of Methyl Anisate

The extraction of anisic acid was conducted by oxidation of K_2_Cr_2_O_7_. Six gram of K_2_Cr_2_O_7_ was dissolved in 30 mL of deionized water and then 6 g of sulfonic type solid acid was slowly added and stirred for further use. One milliliter of anise oil was added in a round bottom flask which was placed in an ice-bath, and then the mixture of K_2_Cr_2_O_7_ and sulfonic type solid acid prepared before was gently added into the solution over 6 h, preventing the rapid oxidation of anise oil resulting in the insufficient oxidation. The solution was under stirring until it changed to light green without obvious oily substances in the upper layer, and then it was heated at 60°C for 2 h without stirring. After cooling to room temperature, the precipitate was collected via filtration and further purified by recrystallization from a mixture of ethanol absolute and deionized water. The obtained anisic acid was a white, acicular crystal product.

Anisic acid can also be extracted by the oxidation of KMnO_4_. The mixture of 7.9 g of KMnO_4_ and 40 mL of deionized water was placed in a round bottom flask while stirring. Five gram of sulfonic type solid acid and 3.1 g of anise oil were added into the solution, separately. The refluxing mixture was heated at 90°C for 6 h and filtered under reduced pressure immediately. The extract was washed with hot deionized water and immersed in an ice-bath and 5.6 mol/L HCl was then added into the solution until the pH value decreased to < pH 3 with the white precipitation. After 2 h, the mixture was extracted and recrystallized from a mixture of ethanol absolute and deionized water. A white, acicular crystal product was obtained as anisic acid.

The third method for extraction of anisic acid was through oxidation of H_2_O_2_. Anise oil (7.5 mL) was placed in 50 mL of round bottom flask while stirring and 1 g of FeCl_3_ was added drop by drop. Heating at 90°C under reflux, 30% of H_2_O_2_ (20 mL) was slowly added into the system over 2 h. After further proceeding for 2 h, the solution was filtered under reduced pressure immediately, washed with hot water three times, immersed in an ice-bath, and 5.6 mol/L of HCl was then added until the solution pH value was < pH 3. During standing, the white solids precipitated out from the solution. The mixture was filtered under reduced pressure and dried to obtain anisic acid as a white, acicular crystallite.

The obtained anisic acid (1.2 g) by KMnO_4_ oxidation method was mixed with sulfonic type solid acid (0.3 g) and methanol (5 g), refluxing at 100°C for 7 h in a round bottom flask. After the reaction, the methanol was removed using a rotary evaporator. After cooling to 50–60°C, the solution was neutralized by the addition of saturated NaCO_3_ and then methyl anisate precipitated out from the solution. The mixture was filtered under reduced pressure distillate and was collected by reduced pressure distillation under 20 mmHg at 160°C which was obtained as methyl anisate.

#### Shikimic Acid Extraction and Methyl Shikimate Preparation

2.1 g of anise oil was added into a round bottom flask refluxing with methanol (250 mL) for 48 h at a boiling point of 80°C. The obtained extract was evaporated to remove methanol, washed three times with hexane and dissolved in deionized water (200 mL). The solution was heated at 80°C for 5 min with addition of formaldehyde (2 mL) and filtered under reduced pressure immediately. The filtrate obtained as a clear amber solution was further purified by recrystallization from a mixture of ethanol absolute and deionized water. The obtained shikimic acid was a yellow, acicular crystal product.

Methyl shikimate can be prepared by SOCl_2_ catalyst. 0.5 g of shikimic acid dissolved in 5 mL of methanol and placed into a 25 mL round bottom flask, immersed in an ice-bath while stirring. 0.5 mL of SOCl_2_ was added dropwise. The reaction mixture was heated under 40°C refluxing for 3 h and then concentrated. The obtained thickened liquid was dissolved in 5 mL of ethyl acetate and self-standing for 10 min after completely mixing. The obtained upper layer was collected by a separatory funnel and then the contents were evaporated to dryness on a watch glass. Methyl shikimate was obtained as a white crystal after cooling.

Another method for preparation of methyl shikimate is through sulfonic type solid acid catalyst. The mixture of 0.5 g of shikimic acid, 0.5 g sulfonic type solid acid and 5 mL of absolute methanol was placed in a 25 mL round bottom flask, refluxing under 40°C for 3 h. the obtained solution was concentrated, completely dissolved in 5 mL of ethyl acetate, and filtered. The filtrate was dried on a watch glass and methyl shikimate as a crystal was obtained.

### Extractives of Black Pepper

#### The Extraction and Purification of Piperine

Ground black pepper fruit (10 g, 60 mesh powder) was dissolved in 100 mL of ethanol absolute, refluxing under 80°C for 2 h. The filtrate and residue were collected separately by vacuum filtration, standing for further use. The residue was dissolved in 100 mL of ethanol absolute, refluxing under the same condition. The filtrate was collected and mixed with the obtained filtrate. The mixture of these two filtrates was concentrated to 10–15 mL by reduced pressure distillation and the pH value was adjusted to pH 4 by 6 mol/L HCl, standing for 4 h. After centrifugation at 1,200 rpm for 10 min and filtration, the precipitate (piperic acid undissolved in acid) was removed and the filtrate was adjusted by 10% NaOH solution to pH 11, standing for 2 h, and then immersed in the hot water bath. The deionized water was slowly added until the solution became turbid and a yellow solid precipitated out from the solution. The mixture was placed in an ice-bath for 4 h and then centrifuged at 1,200 rpm for 10 min. A yellow powdered solid was obtained as the crude piperine.

Acetone (25 mL) was added into the obtained crude piperine, dissolved by gentle heating with or without the precipitate from the solution. Either the solution with precipitate was filtered by heating to obtain the solid or the solution without precipitate was added deionized water (25 mL) until the precipitate come out from the solution and then centrifuged and filtered, repeating 3 times to obtain the solid. The liposoluble compounds in the obtained solid were removed by diethyl ether. The yellow powder was obtained as piperine.

#### Piperic Acid Extraction and Methyl Piperate Preparation

Piperine (3 g) was dissolved in 75 mL of KOH/ethanol (v/v = 1/4) solution in a round bottom flask, refluxing at 80°C for 15 h. The solution was filtered under the reduced pressure and the residue was dissolved in 90 mL of deionized water with gentle heating. The mixture was adjusted by HCl solution to pH 3 and left to stand until the precipitate came out of the solution and filtered under the reduced pressure. The yellow solid was obtained as crude piperic acid. The obtained crude piperic acid was dissolved in 65 mL of benzene and filtered under the reduced pressure. The obtained residue was washed with diethyl ether and dried as piperic acid.

In a 25 mL round bottom flask, piperic acid (0.25 g) dissolved in 10 mL of anhydrous methanol. Sulfonic type solid acid (0.1 g) was added, refluxing for 6 h. The precipitate was collected by reduced pressure distillation, washing with deionized water and saturated NaHCO_3_ solution, separately. After cooling, drying, and recrystallizing by acetone, the methyl piperate was obtained as a yellow crystal.

### Methods

The yield of obtained biomasses is identified by Equation (1):

(1) $$\begin{array}{l} \text{Yield of biomass }\left(\text{\%}\right)=\left({\text{W}}_{\text{t}}/{\text{W}}_{0}\right)\times 100 \end{array}$$

where W_t_ and W_0_ are the weight of obtained biomass and original product, respectively.

The purity of obtained biomasses is identified by Equation (2) via Area Normalization method:

(2) $$\begin{array}{l} \text{Purity of analyte }\left(\text{\%}\right)=\left({\text{A}}_{\text{m}}/{\text{A}}_{\text{n}}\right)\times 100 \end{array}$$

where A_m_ and A_n_ are the peak area of analyte and the total area of all peaks, respectively.

^1^H NMR and ^13^C NMR spectra were recorded on Bruker AM-300 spectrometer operating at 300 MHz and Bruker AM-400 spectrometer at 400 MHz, respectively. Fourier transmission infrared (FTIR) measurements were performed by a Nicolet MAGNA-550 FTIR (Nicolet Instrument Co., Madison, WI) at room temperature. The samples were dried and pressed in a KBr pellet. UV-vis spectra were carried out on a UV-2550 spectrophotometer (Beijing Sartorius Co., Ltd., China). The fluorescence spectra were conducted on a Hitachi F-4500 fluorescence spectrophotometer (Hitachi, Ltd., Tokyo, Japan). High-performance liquid chromatography (HPLC) analysis was carried out on Waters Alliance HPLC system, assembled by Waters 600 controller, Waters 600 pump, and Waters 2996 photodiode array detector (Waters, Milford, MA, USA). The chromatographic separation was performed by injecting a 5 μL sample volume on a C18 column (4.8 × 150 mm, 5 μm particle size).

### Antibacterial Activity Study

Antibacterial activity of the obtained biomasses was evaluated against five common microorganisms. These tested extracts were dissolved in DMF (10 μL/mL) with the highest concentration of 3,200 μg/mL. Serial dilutions of the test extracts were prepared in DMF, and 30 μL of each dilution was added to 3 mL broth. The exponentially growing cells of each microorganism were inoculated into the broth of ~10^5^ cells/mL. After 48 h incubation, minimum inhibitory concentration (MIC) was established as the lowest concentration of the test extracts, demonstrating no visible growth. All experiments were performed in triplicate.

### Antioxidant Activity Study

Antioxidant activity of biomasses was evaluated by DPPH scavenging assay by M. Burits and F. Bucar (Burits and Bucar, 2000). The tested biomass (0.1 ml) at different concentrations (1.0, 2.0, 4.0, 8.0, and 10.0 mg/mL) was added into 5 mL of 0.05%0 DPPH in methanol solution. After incubation for 30 min at room temperature in the dark, the absorbance was read against pure methanol at 520 nm. Inhibitory rate against free radicals DPPH was calculated by Equation (3):

(3) $$\begin{array}{l} \text{Inhibitory rate }\left(\text{\%}\right)=\left[\left({\text{A}}_{\text{c}}-{\text{A}}_{\text{t}}\right)/{\text{A}}_{\text{c}}\right]\times 100 \end{array}$$

where A_c_ and A_t_ are the absorbance of the control and the tested biomass. Values of the biomasses were compared with that of BHT (positive control). All experiments were performed in triplicate.

### Anticancer Profiles

The *in vitro* anticancer activities of the obtained biomasses against C6 cells were determined by MTT [3-(4,5-dimethylthiazol-2-yl)-2,5-diphenyl tetrazolium bromide] assay (Heilmann et al., 2001). Hundred microliter of cell suspension (1 × 10^4^ cells) were seeded into each well of a 96-well microtiter plate, incubating at 37°C overnight. Cells were treated with different concentrations (1.0, 2.0, 4.0, 6.0, 8.0, 10.0, 15.0, 20.0 μg/mL) of samples for 24 h. PBS buffer was used as the medium for the control group. After cultivation, MTT solution (5 mg/mL in 20 μL PBS) was added into each well, followed by incubation at 37°C for another 4 h. The supernatant was removed and 150 μL/well of DMSO was used to dissolve the precipitated formazan. The absorbance value was measured at 570 nm using a microplate reader and the inhibitory rate was calculated. All experiments were performed in quintuplicate. Inhibitory rate of anticancer activity was calculated by Equation (4):

(4) $$\begin{array}{l} \text{Inhibitory rate }\left(\text{\%}\right)=\left[\left({\text{A}}_{0}-{\text{A}}_{1}\right)/{\text{A}}_{0}\right]\times 100 \end{array}$$

where A_0_ and A_1_ are the absorbance of untreated cells and treated cells. All experiments were performed in triplicate.

## Results

### Characterizations of Anise Oil

**Figure 1:**
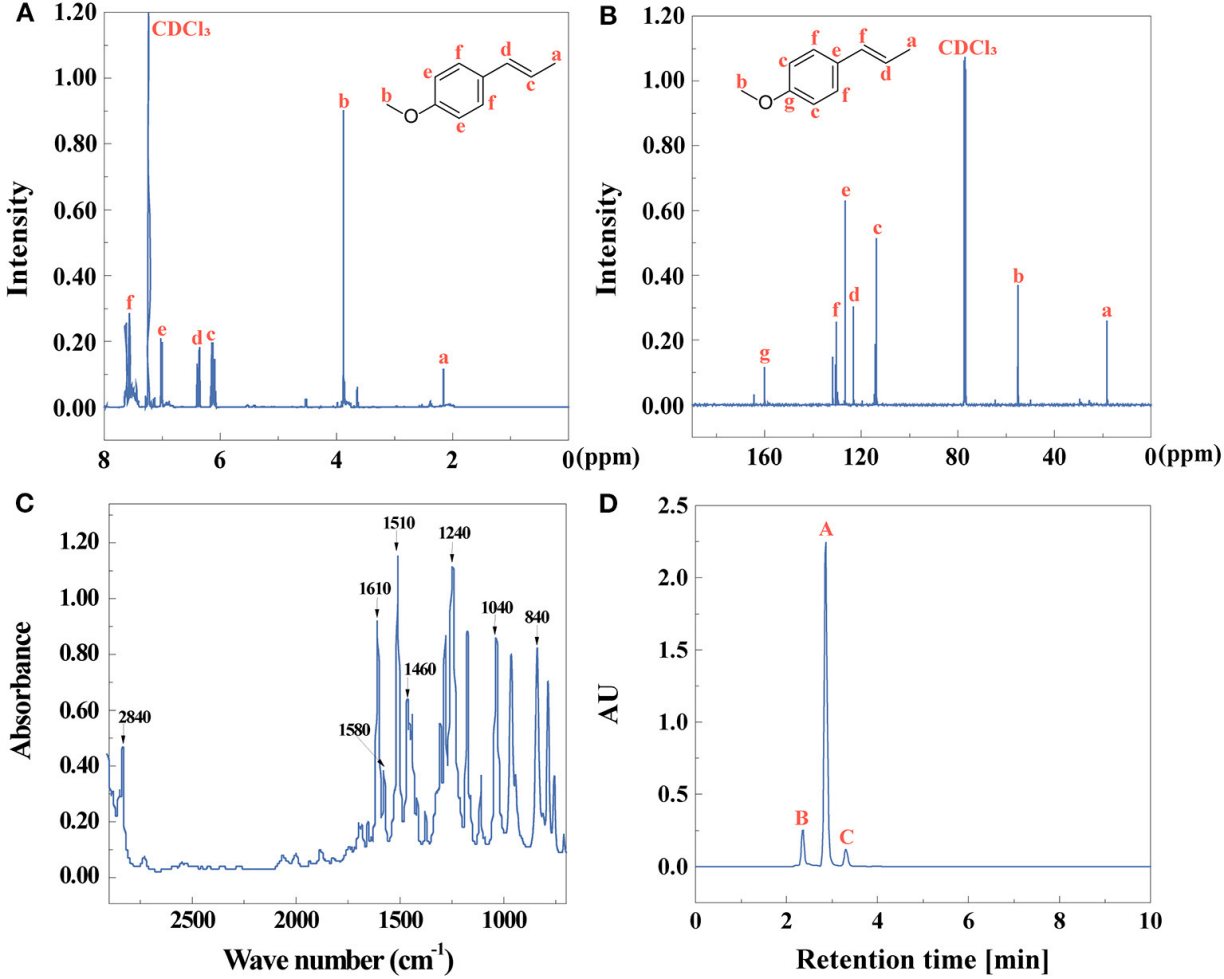
**(A)** ^1^H NMR, **(B)** ^13^C NMR, **(C)** FTIR spectra, and **(D)** HPLC (peak **A**: anise oil, peak **B,C**: other compounds) of anise oil.

After the first extractive step, the chemical composition and purity of extractives of star anise were identified by ^1^H NMR, ^13^C NMR, FTIR, and HPLC as shown in Figure 1. Figures 1A,B shows the ^1^H NMR and ^13^C NMR spectra of star anise, respectively. The FTIR spectrum further confirmed the structures in Figure 1C. The characteristic peaks at 1,580, 1,510, and 1,460 cm^−1^ attributed to skeletal vibrations of a benzene ring, and the peak at 3,020 cm^−1^ was assigned to C-H aromatic stretching mode, indicating the existence of benzene ring (Zhang et al., 2018). The C-H out-of-plane bending vibrations are related to the substituent aromatics assigned at 840 cm^−1^ (Suhem et al., 2015). The peaks at 1,240 and 1,040 cm^−1^ ascribed to C-O-C stretching vibrations (Ghazy et al., 2021). The peak at 2,840 and 1,610 cm^−1^ indicated the existence of O-CH_3_ and C=C (Hoque et al., 2011). These results show that the main component of anise oil is p-propenylanisole (anethole). The yield and purity (Figure 1D) of anise oil is 87 and 83%, identified by Equations (1, 2), respectively.

### Characterizations of Anisic Acid and Methyl Anisate

**Figure 2:**
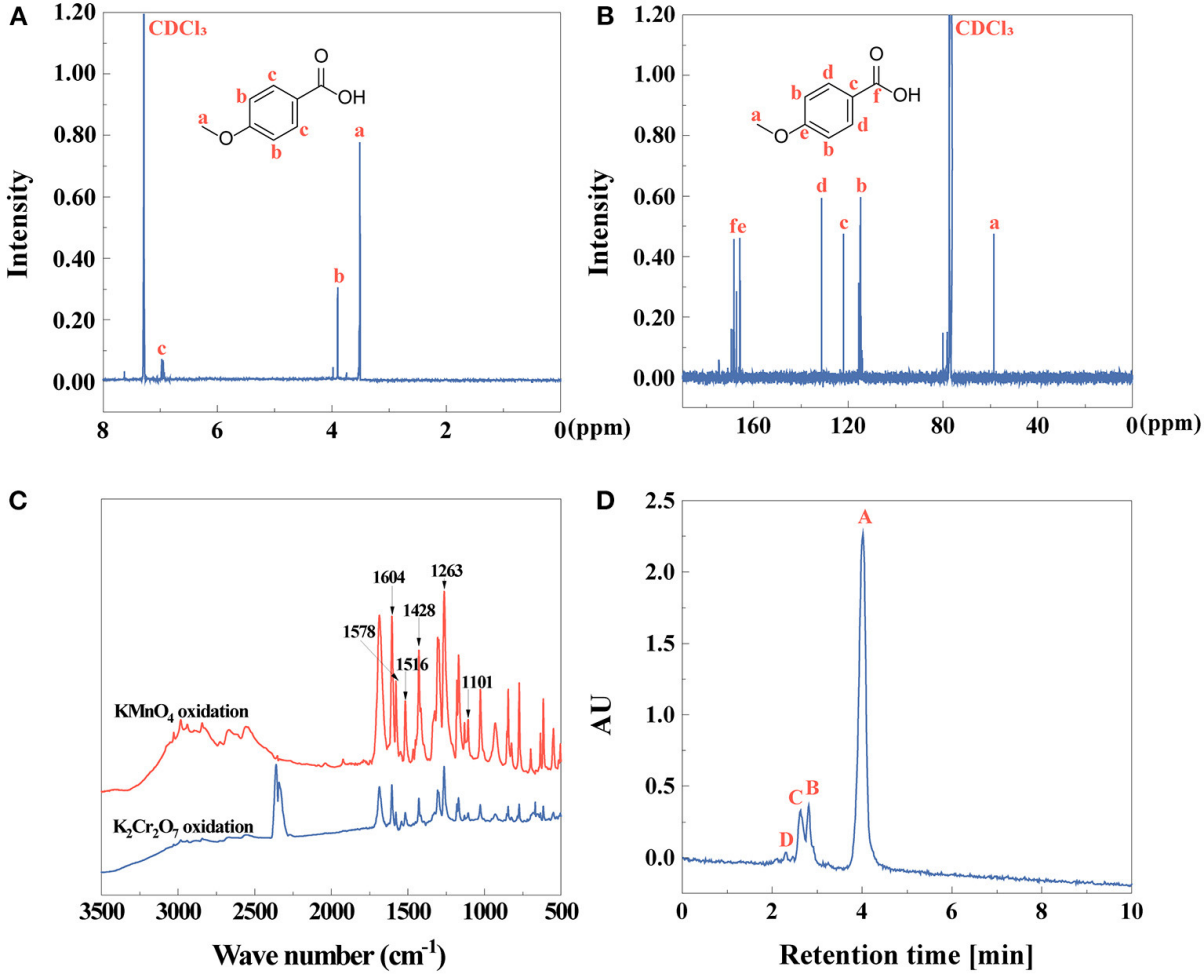
**(A)** ^1^H NMR, **(B)** ^13^C NMR, **(C)** FTIR spectra, and **(D)** HPLC (peak **A**: anisic acid, peak **B–D**: other compounds) of anisic acid.

After obtaining anise oil, anisic acid has been shown to be an essential compound after the second step of extraction. The chemical composition of the extracts were identified by ^1^H NMR, ^13^C NMR, FTIR spectra, HPLC (Figure 2) and fluorescence spectrometry (Supplementary Figure 1A). The successful extraction of anisic acid by KMnO_4_ oxidation was confirmed by ^1^H NMR and ^13^C NMR spectra (Figures 2A,B). The FTIR spectra characterized anisic acid depending on the oxidative-extraction methods by K_2_Cr_2_O_7_ and KMnO_4_, separately (Figure 2C). Comparing these two spectra, it is indicated that anisic acid oxidized by KMnO_4_ showed a high purity due to the characteristic peaks than that oxidized by K_2_Cr_2_O_7_ because of the impurities on the characteristics of anisic acid. Therefore, the FTIR spectrum of anisic acid by KMnO_4_ was analyzed. The characteristic peaks at 1,604, 1,578, 1,516, 1,428 cm^−1^ appeared in the FTIR spectrum were assigned to the skeletal vibrations of the benzene ring (Sardjono et al., 2012). The peaks at 1,263 and 1,101 cm^−1^ were attributed to C-O-C stretching vibrations, indicating the existence of aromatic ether (Jia et al., 2020). The stretching vibration of C=O is 1,686 cm^−1^ (Ngilirabanga et al., 2020). All these characteristic peaks proved that p-methoxybenzoic acid (anisic acid) was obtained. In Supplementary Figure 1A, the fluorescent properties of anisic acid were studied. In fluorescence spectra, the intensity of maximum excitation peak is at 279 nm and the emission peak was observed at 305 nm. The yield of anisic acid by KMnO_4_ or K_2_Cr_2_O_7_ oxidation is 75 or 41% by Equation (1), respectively. The purity of anisic acid (Figure 2D) by KMnO_4_ oxidation is 82% identified by Equation (2).

**Figure 3:**
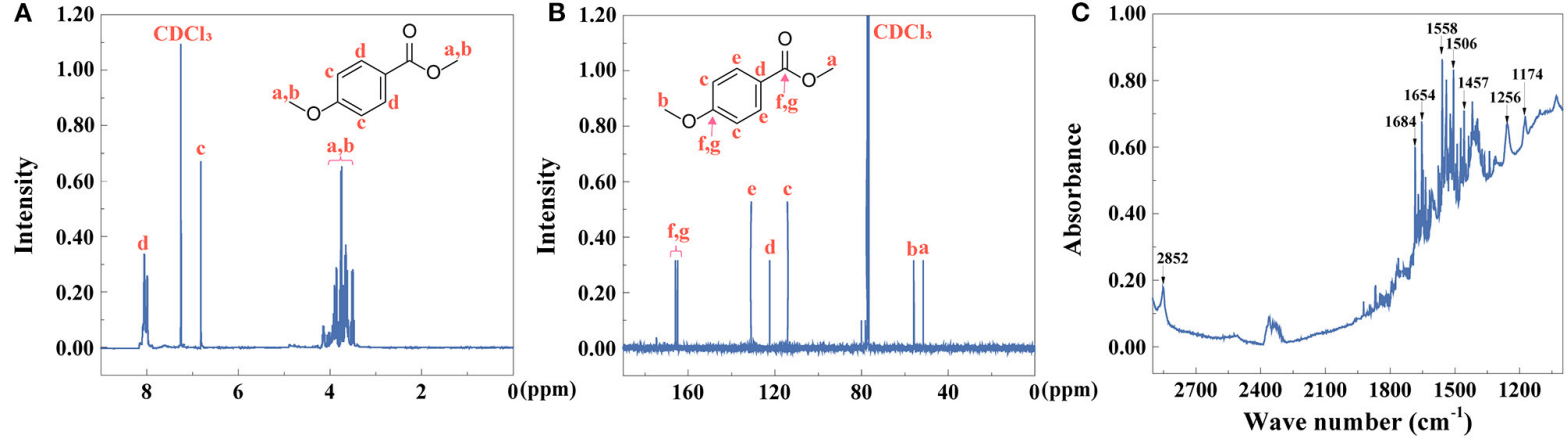
**(A)** ^1^H NMR, **(B)** ^13^C NMR, and **(C)** FTIR spectra of methyl anisate.

Methyl anisate was synthesized by esterification of anisic acid and characterized in Figure 3. ^1^H NMR and ^13^C NMR spectra of methyl anisate is given in Figures 3A,B, respectively. The FTIR spectrum of methyl anisate in Figure 3C exhibits the absorption peaks at 2,852 and 1,684 cm^−1^ attributed to O-CH_3_ stretching vibration and C=O stretching vibration, separately (Mathammal et al., 2013). The skeletal vibrations of the benzene ring were at 1,654, 1,558, 1,506, 1,457 cm^−1^ (Jeyakumar and Narayanasamy, 2020). The peak 1,256 cm^−1^ was attributed to aromatic ether (Szymulanska-Ramamurthy et al., 2017). The asymmetric stretching vibration of C-O-C was at 1,174 cm^−1^ (Dehghani et al., 2020). It is obvious that methyl anisate was obtained. The fluorescence spectrum of methyl anisate was shown in Supplementary Figure 1B. The strongest excitation wavelength was placed at 355 nm. While 355 nm was chosen as the operating excitation wavelength, the emission peak is at 374 nm. The yield of methyl anisate is 63% by Equation (1).

### Characterizations of Shikimic Acid and Methyl Shikimate

**Figure 4:**
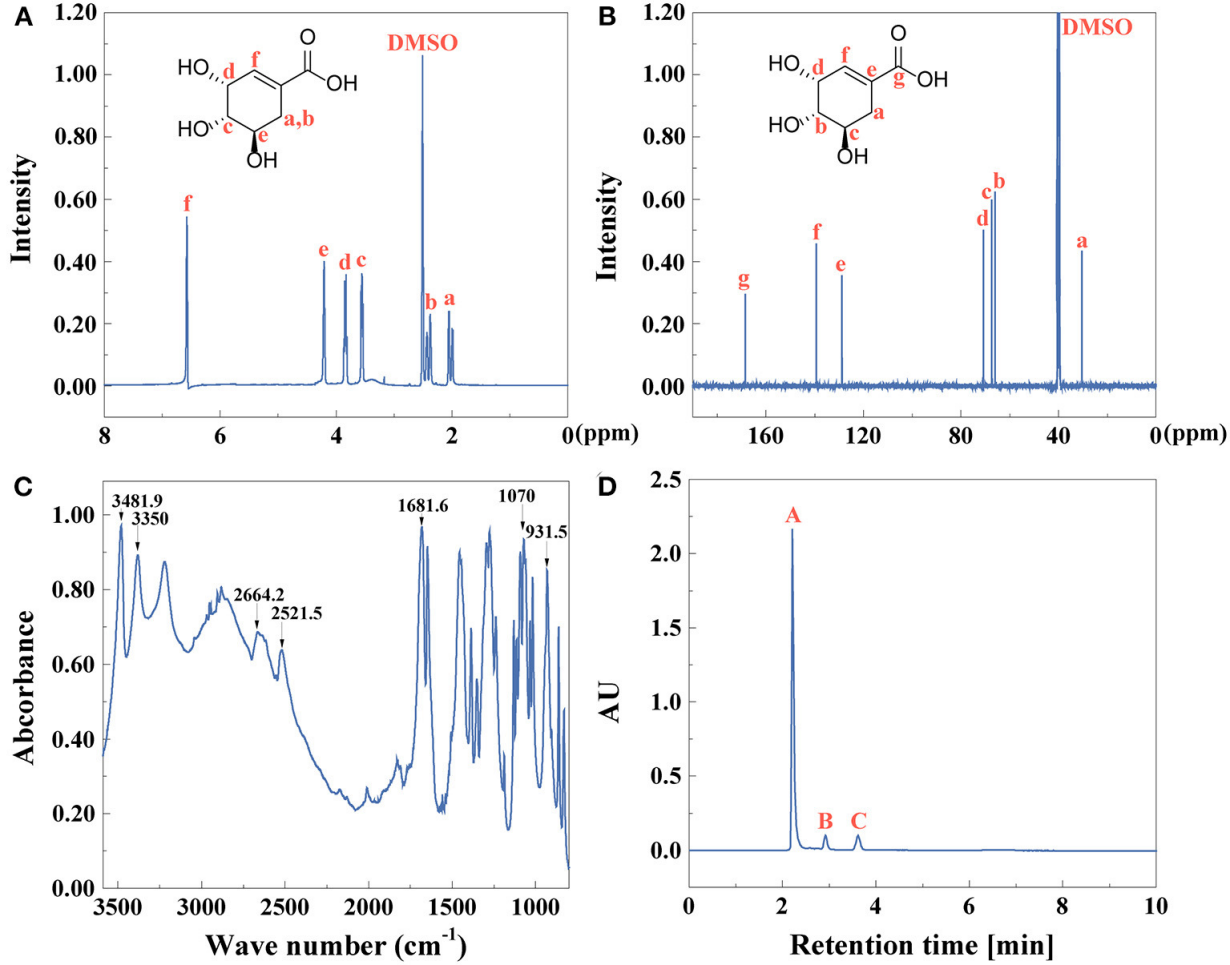
**(A)** ^1^H NMR, **(B)** ^13^C NMR, **(C)** FTIR spectra, and **(D)** HPLC (peak **A**: shikimic acid, peak **B,C**: other compounds) of shikimic acid.

After extracting anise oil, shikimic acid was then extracted and identified in Figure 4. ^1^H NMR and ^13^C NMR spectra of shikimic acid were recorded as shown in Figures 4A,B, respectively. The FTIR spectrum of shikimic acid was shown in Figure 4C. The bands at 3481.9 cm^−1^ referred to stretching vibration of association O-H group. The free O-H in carboxy group was shown at 3,350 cm^−1^ (Pavinatto et al., 2020). If the dimer was formed, the peak of O-H would shift to a lower range at 2664.2 and 2521.5 cm^−1^. The peak of C=C in alkene was at 1681.6 cm^−1^. The characteristic peak of carboxylic acid was at 931.5 cm^−1^ and the peak at 1,070 cm^−1^ was assigned to stretching vibration of C-O (Rawat et al., 2013).

UV-vis spectra indicated that the absorption peak of shikimic acid was at 205 nm due to the conjugated double bonds C=C-C=O (Supplementary Figure 2A). As shown in Supplementary Figure 2B, the fluorescence spectrum of shikimic acid was investigated. With the excitation wavelength at 235 nm, the emission wavelength was at 346 nm. The yield of shikimic acid is 79%, identified by Equation (1). The purity of shikimic acid (Figure 4D) is 85% identified by Equation (2).

**Figure 5:**
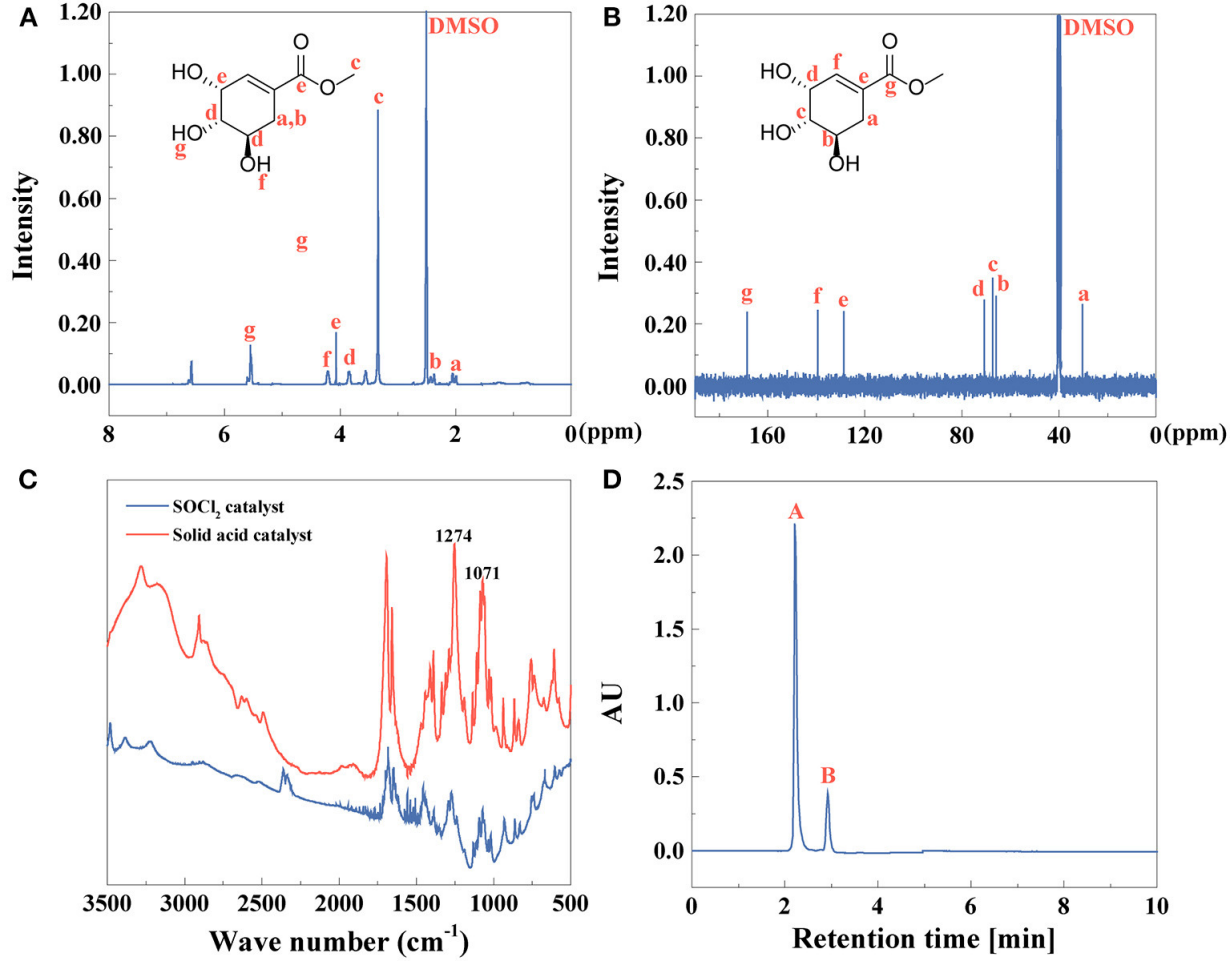
**(A)** ^1^H NMR, **(B)** ^13^C NMR, **(C)** FTIR spectra, and **(D)** HPLC (peak **A**: methyl shikimate, peak **B**: other compounds) of methyl shikimate.

By esterification of shikimic acid, methyl shikimate was obtained, identified, and quantified as shown in Figure 5. ^1^H NMR and ^13^C NMR spectra of methyl shikimate by solid acid catalyst were observed in Figures 5A,B. FTIR spectrum of methyl shikimate catalyzed by SOCl_2_ or solid acid was shown in Figure 5C. Compared with FTIR spectrum of shikimic acid (Figure 4C), O-H of carboxyl at 3,000–2,500 cm^−1^ becomes weaker. The peaks at 3,100 3,000 cm^−1^ or 1,680 1,620 cm^−1^ were assigned to C-H or C=C of alkene (Andayany et al., 2019). The peaks at 1,071 and 1,058 cm^−1^ or 1,274 and 1,253.5 cm^−1^ were ascribed to symmetrical stretching vibration or asymmetrical stretching vibration of C-O-C (Yeoh et al., 2015). These results indicated that the methyl shikimate was obtained by the two catalysts.

UV-vis spectrum of methyl shikimate indicated that the absorption peak is blue-shifted to 201 nm (Supplementary Figure 3A) compared to shikimic acid at 205 nm (Supplementary Figure 2A) due to the conjugated double bonds C=C-C=O. The fluorescence spectra of methyl shikimate catalyzed by both SOCl_2_ and solid acid were shown in Supplementary Figures 3B,C, respectively. The excitation wavelengths of these two samples were 232 and 234 nm, and the emission wavelengths of these two samples were 340 nm (Supplementary Figure 3B) and 349 nm (Supplementary Figure 3C), respectively. The yield of methyl shikimate is 63%, identified by Equation (1). The purity of methyl shikimate (Figure 5D) is 82% identified by Equation (2).

### Bioactivity Studies of the Biomasses Obtained From Star Anise

#### Antibacterial Activity of Anise Oil, Anisic Acid, and Shikimic Acid

As shown in Table 1, the antibacterial activities of anise oil, anisic acid, and shikimic acid were evaluated against two Gram-positive bacteria [*S.aureus* (MRSA), *S.pyogenes*], and three Gram-negative bacteria (*E.coli, S.typhi, P.aeruginosa*). Anisic acid and shikimic acid exhibited pronounced activity against microorganism than anise oil. Anisic acid showed higher antibacterial activity against *S.aureus, S.pyogenes*, and *S.typhi*, whereas shikimic acid exhibited higher antibacterial activity against *S.pyogenes, E.coli*, and *S.typhi*. The antibacterial efficacy of anisic acid and shikimic acid may be due to their anionic carboxylic head group, similar to that of weak-acid preservatives, e.g., benzoic acid and sorbic acid. It is indicated that this highly hydrated head group enabled association with the water-lipid interface of the membrane, and thus its passage through the lipid core was impermeable due to a high-energy barrier. Therefore, in growing cells, the major energy was required for displacing the equilibrium, resulting in a low growth (Cole and Keenan, 1987; Warth, 1988). The results indicated that two star anise extractives exhibit antimicrobial activities and thus can be considered as a drug candidate in the treatment of infectious diseases caused by the tested pathogenic microbes.

**Table 1:** Antibacterial activity of star anise extractives.

**MIC μg/ml tested compounds**	**Microorganism tested**				
	***S.aureus* (MRSA)**	***S.pyogenes***	***E.coli***	***S.typhi***	***P.aeruginosa***
Anise oil	1,200	1,600	1,600	1,000	1,600
Anisic acid	800	400	1,200	800	2,000
Shikimic acid	1,200	800	800	400	1,600

Moreover, compared to nanomaterials previously reported as antibacterial agents, the biomasses obtained from star anise show a similar antibacterial activity as these nanomaterials, such as sulfonated chitosan-based antibiofilm (Huang et al., 2019) and natural clay nanocomposites (Dong et al., 2020). However, star anise extractives exhibit lower antibacterial activities than that of nanocomposites (TiO_2_/Sb_2_S_3_/GQDs) mixed with graphene quantum dots (GQD) (Teymourinia et al., 2019), but display higher antibacterial efficacy than that of ultra-small copper oxide (II) nanoparticles (Moniri Javadhesari et al., 2019). Therefore, it is indicated that the obtained extractives of star anise can be performed as the antibacterial agents in the biomedical fields.

#### Antioxidant Activity of Anise Oil, Anisic Acid, Methyl Anisate, Shikimic Acid, and Methyl Shikimate

**Figure 6:**
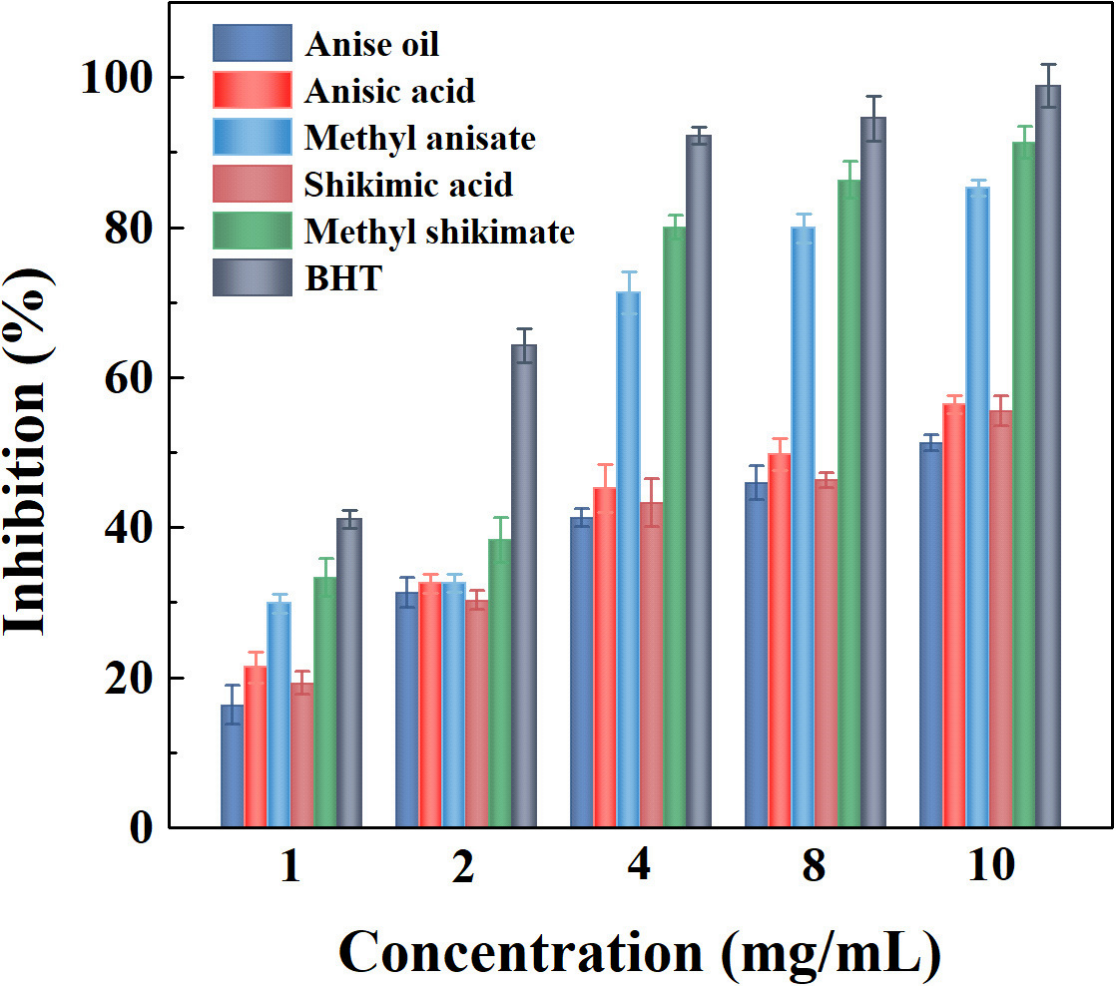
Antioxidant efficacy of anise oil, anisic acid, methyl anisate, shikimic acid, and methyl shikimate.

For food products, antioxidant properties can be used as an indicator for enhancing the shelf-life of foods, e.g., spices (Prakash et al., 2011). Therefore, the free radical-scavenging activities determined by Equation (3) of these five derivatives of star anise were evaluated in comparison with BHT using a DPPH assay as shown in Figure 6 and Table 2. The results showed that the antioxidant activity was dose-dependent for each sample. The three extractives, anise oil, anisic acid, and shikimic acid, provided slight antioxidant activities compared to BHT. As the methanol preparation extractive, methyl anisate, possessed significant antioxidant activities. Methyl shikimate, the ethyl acetate extractive, showed the highest antioxidant activities among all extracts of star anise. The high antioxidant activity of methanol preparation extractives may be because the compounds extracted in methanol solvents have high capability for donating electrons to free radicals, indicating the radical scavenging activities (Maiti et al., 2014). These two compounds can be used as natural antioxidants for enhancing the shelf-life of food.

**Table 2:** IC_50_ value of tested compounds.

**Compound tested**	**Anise oil**	**Anisic acid**	**Methyl anisate**	**Shikimic**	**Methyl acid**	**BHT shikimate**
IC_50_ (mg/mL)	9.88	8.04	2.90	8.96	2.44	1.32

### Characterization of Piperine

**Figure 7:**
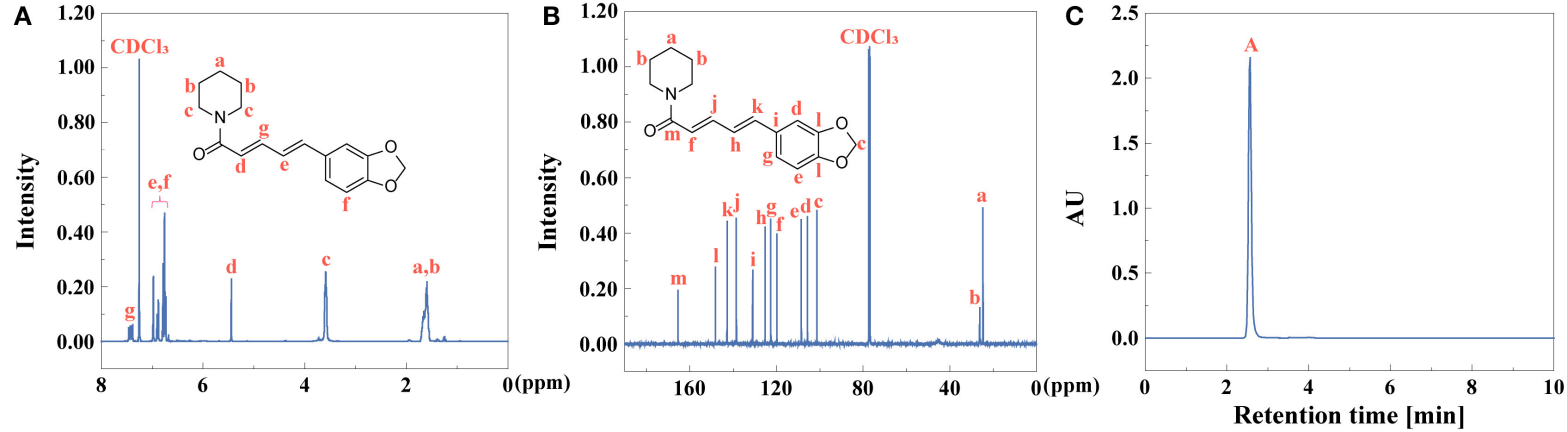
**(A)** ^1^H NMR, **(B)** ^13^C NMR, and **(C)** HPLC (peak **A**: piperine) of piperine.

As the first extractive step of black pepper, the yield of piperine is calculated to be 79%, identified by Equation (1). ^1^H NMR and ^13^C NMR spectra of piperine were collected as shown in Figures 7A,B. UV-vis spectra of piperine showed that absorption peak was at 343 nm (Supplementary Figure 4A). The fluorescent property of piperine was investigated (Supplementary Figure 4B). The fluorescence excitation-emission wavelengths were at 270 and 349 nm, respectively. The purity of piperine (Figure 7C) is above 99% identified by Equation (2).

### Characterization of Piperic Acid and Methyl Piperate

**Figure 8:**
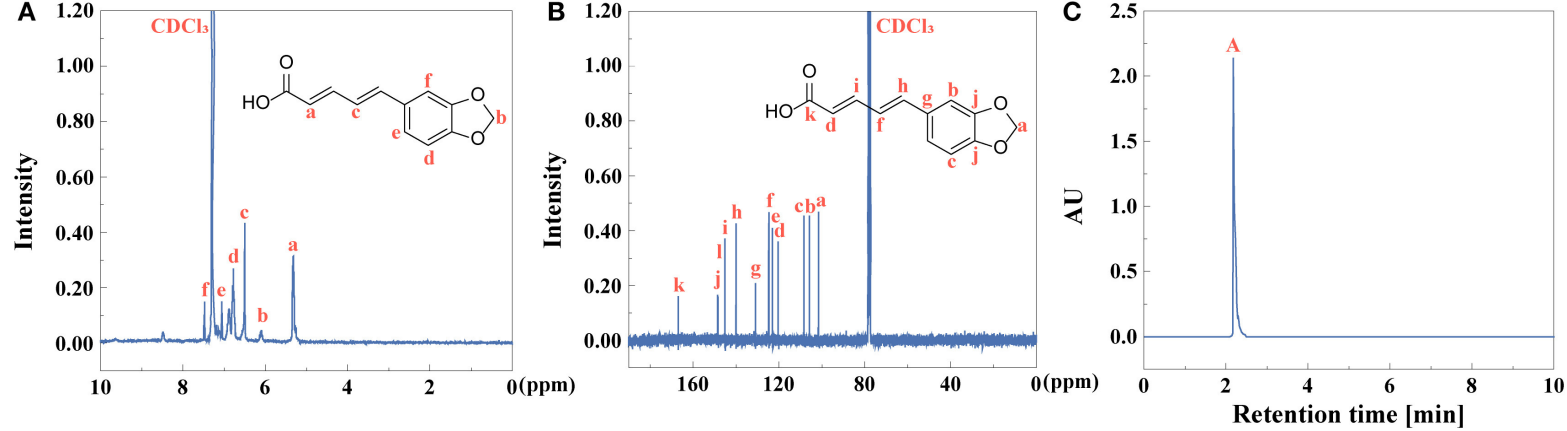
**(A)** ^1^H NMR, **(B)** ^13^C NMR, and **(C)** HPLC (peak **A**: piperic acid) of piperic acid.

After extraction of piperine, piperic acid and methyl piperate were extracted and prepared. The yield of piperic acid and methyl piperate is 72 and 61%, identified by Equation (1). ^1^H NMR and ^13^C NMR spectra of piperic acid were observed in Figures 8A,B, respectively. UV-vis spectrum of piperic acid was shown in Supplementary Figure 5A. The absorption peak is at 336 nm due to the conjugated system with different chromogenic and auxochrome groups. In Supplementary Figure 5B, the fluorescence spectrum of piperic acid show that the excitation and emission peaks are at 349 and 446 nm, respectively.

**Figure 9:**
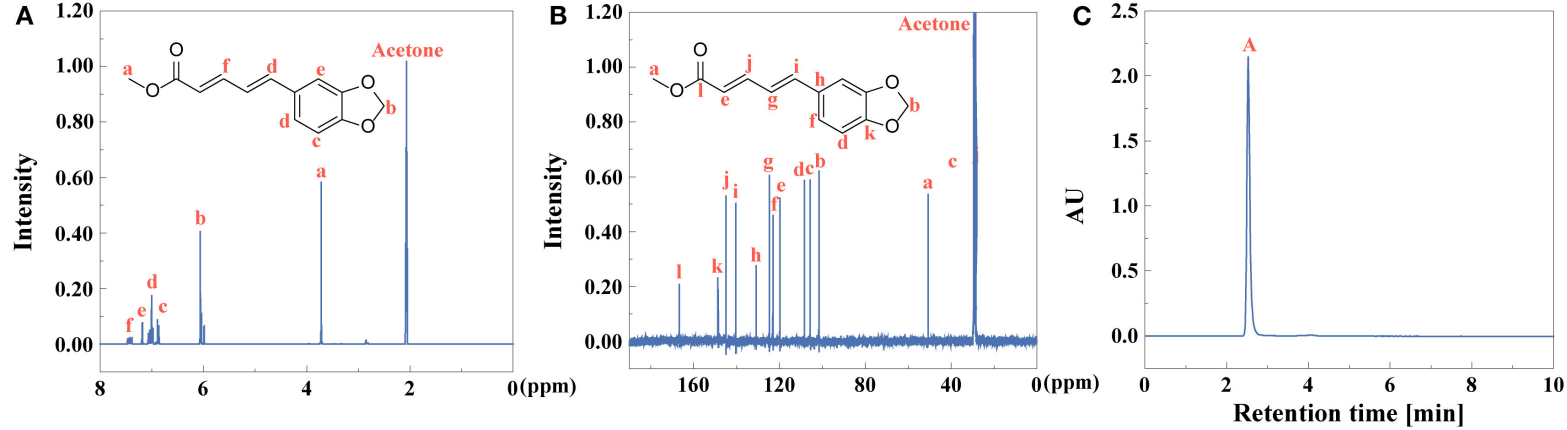
**(A)** ^1^H NMR, **(B)** ^13^C NMR, and **(C)** HPLC (peak **A**: methyl piperate) of methyl piperate.

^1^H NMR and ^13^C NMR spectra of methyl piperate were observed in Figures 9A,B. In Supplementary Figure 5C, UV-vis spectrum of methyl piperate was shown and the absorption peak was at 341 nm. As shown in Supplementary Figure 5D, the fluorescence spectrum of methyl piperate show that the excitation and emission peaks are at 268 and 369.4 nm, respectively. The purity of piperic acid (Figure 8C) and methyl piperate (Figure 9C) are above 99 and 99% identified by Equation (2), respectively.

### Bioactivity Studies of Piperine and Methyl Piperate

#### Anticancer Activities of Piperine and Methyl Piperate

**Figure 10:**
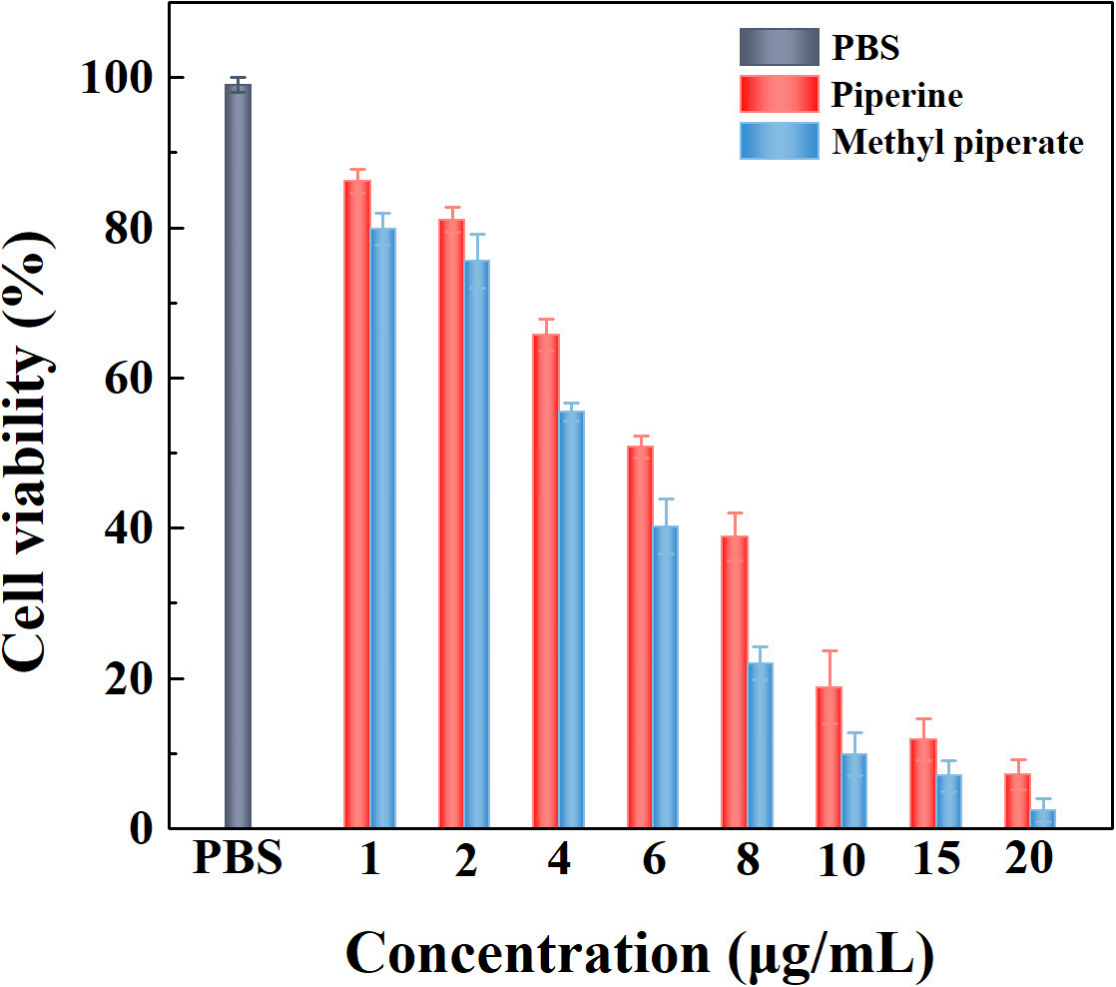
The cell inhibition of C6 cells treated with different piperine and methyl piperate concentrations.

The anticancer activities *in vitro* were evaluated by MTT assay in terms of Equation (4) determined by treating C6 cells with piperine and methyl piperate. The results show that the anticancer activities increased with the enhancement of piperine or methyl piperate concentrations, exhibiting dose-dependent behaviors (Figure 10). The IC_50_ of piperine and methyl piperate is 6.23 μg/mL and 5.54 μg/mL, indicating that both piperine and methyl piperate enable a good inhibition of the growth of cancer cells. After the treatment in cancer cells, the pepper alkaloids presenting in black pepper extractives could eventually induce apoptosis, applied as anticancer agents (Sriwiriyajan et al., 2016; Tiwari et al., 2020). The cells treated with methyl piperate showed a higher inhibition rate than that with piperine.

#### Antibacterial Activity of Piperine and Methyl Piperate

The antibacterial activity of piperine and methyl piperate was evaluated against five different microorganisms as shown in Table 3. The tested compounds showed no obvious antibacterial activity against *S.aureus* (MRSA), *S.pyogenes, S.typhi*, and *P.aeruginosa*, but did show a significant activity against *E.coli*, indicating that the antibacterial activity of black pepper extracts is highly dependent on the type of microorganisms. Phytochemicals present in black pepper extractives, e.g., piperine and methyl piperate, contribute to the antimicrobial potential (Butt et al., 2013). The results indicated that the extractives of black pepper demonstrated dual activities against both microbial infections and cancer.

**Table 3:** Antibacterial activity of piperine and methyl piperate.

**MIC μg/ml tested compounds**	**Microorganism tested**				
	***S.aureus (MRSA)***	***S.pyogenes***	***E.coli***	***S.typhi***	***P.aeruginosa***
Piperine	1,600	1,200	400	1,200	1,600
Methyl piperate	2,000	2,400	800	2,000	2,400

## Conclusions

In the proposed extraction methods, we have demonstrated that the extraction and quantification of pharmaceutically relevant components from natural plants can be achieved. In a single two-stage multistage extraction-esterification method using steam distilled- or oxidative-extraction process, eight different ingredients were successfully obtained from these two traditional spices.

The results revealed that these biomasses of star anise contained anise oil (87% in yield) in the first-stage, and anisic acid (75% in yield), methyl anisate (63% in yield), shikimic acid (79% in yield), methyl shikimate (71% in yield) in the second-stage, and the bioactive components of black pepper included high yields of piperine (79% in yield) in the first-stage, piperic acid (72% in yield) and methyl piperate (61% in yield) in the second-stage. These bioactive extracts were identified by ^1^H NMR, ^13^C NMR, FTIR, UV-vis, fluorescence spectroscopy, and HPLC. Moreover, the extractives of star anise display high antibacterial activity and antioxidant activities. The extractives of black pepper exhibit antibacterial activity against *E.coli* and good anticancer activity that induces significant cancer cell apoptosis.

The analytical result suggested that the rich bioactive components extracted from star anise or black pepper were obtained. Each component from the previous extraction stage can be also applied as the resource in the next extraction stage, realizing the reproducible utilization of the natural spices. This extraction pattern applied the potential for recycling resources. Moreover, the diverse extractives showed the significant antibacterial, antioxidant, and anticancer activities, providing a great potential for newer food supplements as well as drug species.

## Data Availability Statement

The raw data supporting the conclusions of this article will be made available by the authors, without undue reservation.

## Author Contributions

XL and HC: conceptualization. YL and YM: methodology. HL: investigation and data curation. HL, XW, and XC: writing—original draft preparation. XL, HL, and HC: writing—review and editing. HC: supervision. YL and HC: project administration and funding acquisition. All authors have read and agreed to the published version of the manuscript.

## Conflict of Interest

The authors declare that the research was conducted in the absence of any commercial or financial relationships that could be construed as a potential conflict of interest.
