# Supplementary material for: Multistage Extraction of Star Anise and Black Pepper Derivatives for Antibacterial, Antioxidant, and Anticancer Activity
Source: Front Chem. 2021 May 14;9:660138. doi: 10.3389/fchem.2021.660138 (PMC8160366; doi:10.3389/fchem.2021.660138)
Supplement: Supplementary file 1 [file Data_Sheet_1.pdf]

Supplementary Material

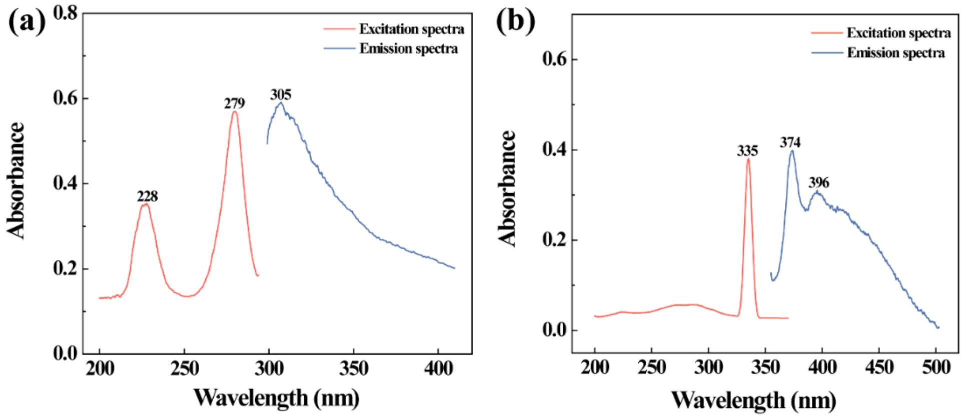

Supplementary Figure 1. (a) The fluorescence spectra of anisic acid and (b) methyl anisate.

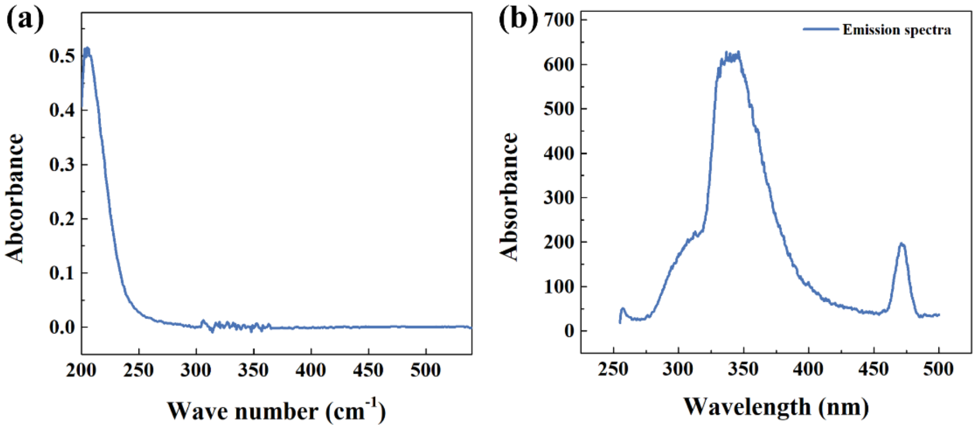

Supplementary Figure 2. (a) UV-vis spectrum and (b) fluorescence spectrum of shikimic acid.

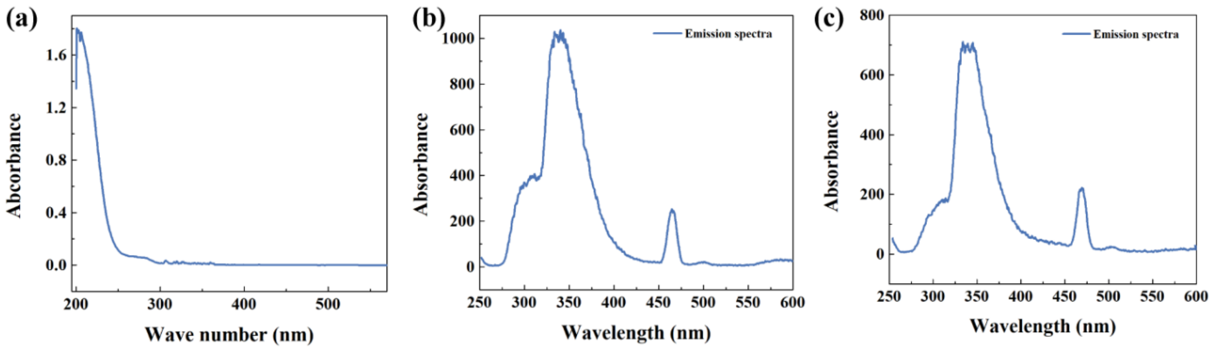

Supplementary Figure 3. (a) UV-vis spectrum of methyl shikimate. The fluorescence spectra of methyl shikimate catalyzed by both $SOCl_2$ (b) and solid acid (c).

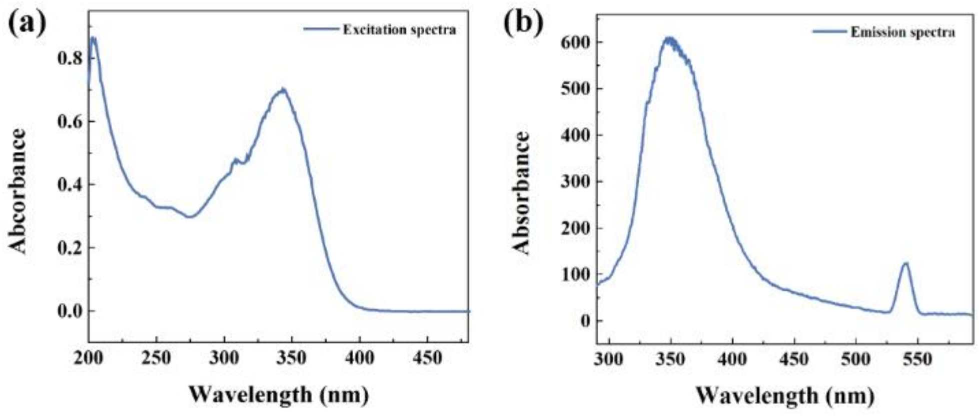

Supplementary Figure 4. (a) UV-vis spectrum and (b) fluorescence spectrum of piperine.

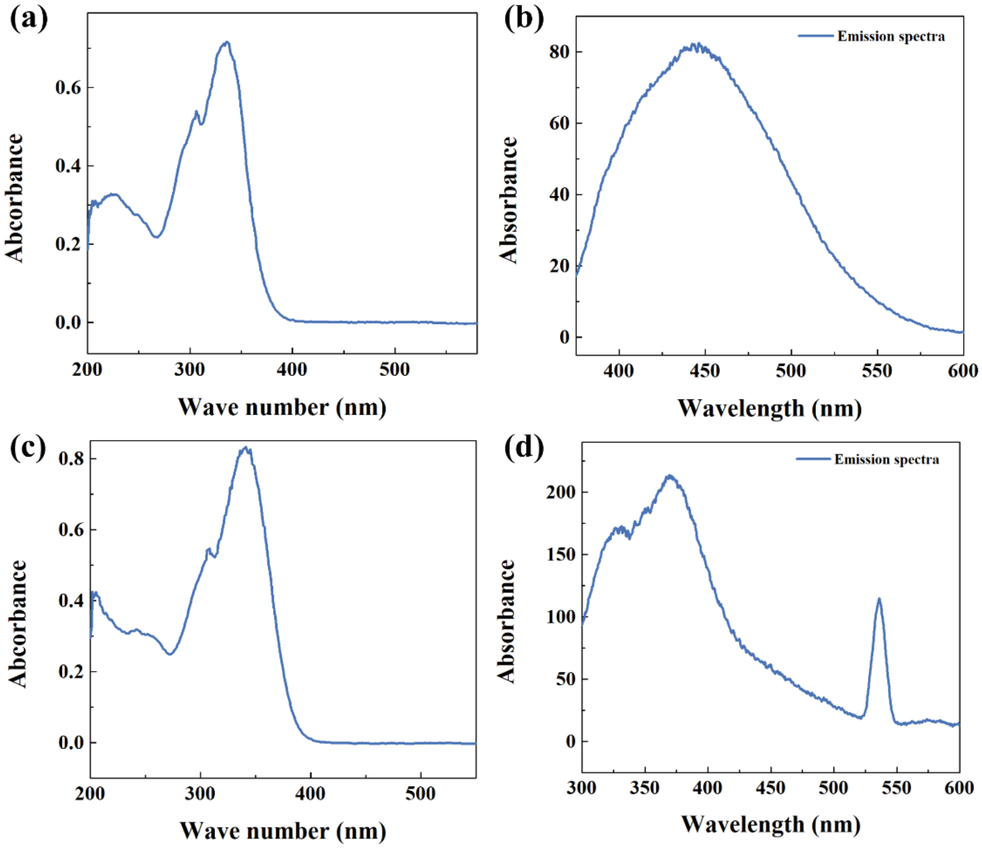

Supplementary Figure 5. (a) UV-vis spectrum and (b) fluorescence spectrum of piperic acid. (c) UV-vis spectrum and (d) fluorescence spectrum of methyl piperate.
